# Value of Multi-Parameter Combined Application of CZT-SPECT and CCTA in Risk Stratification of Short-Term Myocardial Ischemic Events in Patients with Chronic Coronary Syndromes

**DOI:** 10.3390/jcdd13090421

**Published:** 2026-08-28

**Authors:** Jing Ni, Zekun Pang, Haoran Guo, Ajay Kumar Chaudhary, Fukai Zhao, Yue Chen, Jiao Wang, Jianming Li

**Affiliations:** 1 Clinical College of Cardiovascular Diseases, Tianjin Medical University, Tianjin 300457, China; 2 Department of Nuclear Medicine, TEDA International Cardiovascular Hospital, Tianjin 300457, China

**Keywords:** cadmium–zinc–telluride (CZT), single-photon emission computed tomography (SPECT), myocardial perfusion imaging (MPI), coronary computed tomography angiography (CCTA), chronic coronary syndromes (CCS), prognosis

## Abstract

Background: This study aimed to investigate the predictive value of the multi-parameter combined application of cadmium–zinc–telluride single-photon emission computed tomography (CZT-SPECT) and coronary computed tomography angiography (CCTA) for short-term myocardial ischemic events in patients with chronic coronary syndromes (CCS). Methods: A total of 205 CCS patients who underwent both CZT-SPECT and concurrent CCTA were retrospectively enrolled. A reversible ischemia indicator (summed difference score, SDS) from CZT-SPECT and an inflammatory marker (fat attenuation index, FAI) from CCTA were obtained. Short-term myocardial ischemic major adverse cardiovascular events (MACEs) were recorded. Cox regression assessed independent predictive value, while net reclassification improvement (NRI), integrated discrimination improvement (IDI), and Kaplan–Meier curves evaluated incremental predictive and risk stratification ability. Results: 19 myocardial ischemic MACEs occurred. SDS was an independent predictor (HR: 1.386; 95%CI: 1.032–1.861; *p* = 0.030). Adding FAI to the combination of clinical factors and SDS significantly improved prediction (NRI = 0.215, *p* = 0.037; IDI = 0.043, *p* = 0.017). The high-SDS plus high-FAI group had the highest event rates (20.0%, all *p* < 0.05). Conclusions: SDS appears to be an independent predictor of short-term ischemic MACE. FAI may further stratify risk among patients with high SDS, with those exhibiting both high SDS and high FAI having the highest event rates. These findings should be considered hypothesis-generating and warrant confirmation in larger prospective cohorts.

## 1. Introduction

Currently, cardiovascular disease (CVD) remains the leading cause of death worldwide. The Global Burden of CVD Study [1] reported that in 2022, approximately 9.23 million patients died from ischemic heart disease (IHD), with an age-standardized disability-adjusted life years (DALYs) rate of 2275.9 per 100,000 population, ranking first among all CVDs. IHD, also known as coronary artery disease (CAD), is generally defined as a clinical syndrome characterized by myocardial ischemia, hypoxia, or even infarction resulting from coronary atherosclerosis (leading to luminal stenosis or plaque formation) and/or functional alterations (such as vasospasm and microvascular dysfunction), and it is classified into acute coronary syndromes (ACS) and chronic coronary syndromes (CCS) [2,3]. Among CAD types, CCS is the most common. It can lead to major adverse cardiovascular events (MACEs), such as recurrent angina, repeat revascularization, heart attack, or even sudden death [4,5].

Single-photon emission computed tomography (SPECT) myocardial perfusion imaging (MPI) and coronary computed tomography angiography (CCTA) play important roles in the diagnosis, guidance of treatment decisions, and prognostic evaluation of CCS, and have been widely recognized [6,7,8,9]. Among these, the new generation of cardiac-dedicated SPECT based on cadmium–zinc–telluride solid-state semiconductor detector technology (CZT-SPECT) not only significantly improves acquisition sensitivity, shortens acquisition time, and reduces patient radiation dose, but also enables non-invasive quantitative assessment of myocardial blood flow through dynamic MPI (DMPI). Its clinical application is growing increasingly [10,11]. As a convenient non-invasive imaging technique for the screening and diagnosis of CCS, CCTA can clearly visualize the coronary artery lumen and atherosclerotic plaques. Furthermore, with the aid of artificial intelligence (AI)-derived coronary parameters, it further deepens the understanding of cardiovascular risk prediction in patients with CCS [12,13].

With the increasing clinical use of both modalities and the growing number of patients undergoing simultaneous assessment, it is particularly important to integrate their multiparametric information and leverage their respective advantages to improve diagnostic and prognostic accuracy in CCS patients. However, research in this area is currently lacking. Therefore, this study aims to predict short-term myocardial ischemic events in CCS patients through the combined application of cardiac-dedicated CZT-SPECT DMPI and CCTA, and to explore the incremental predictive and risk stratification value of their combined use, thereby providing a more accurate non-invasive risk assessment tool for clinical practice.

## 2. Materials and Methods

### 2.1. Study Design

Clinical and imaging data of patients with suspected or known CCS who successfully underwent cardiac-dedicated CZT-SPECT DMPI and CCTA examinations at our hospital from January 2024 to April 2025 were retrospectively collected. Following the 2019 ESC guidelines, CCS encompasses a spectrum of clinical scenarios, including patients with stable anginal symptoms and/or dyspnea, and asymptomatic individuals with known CAD [14]. Inclusion criteria were age ≥ 18 years; interval between CCTA and CZT-SPECT < 3 months, with no revascularization during this period; complete clinical and imaging data. Exclusion criteria included: prior revascularization; history of heart failure or myocardial infarction; severe arrhythmia (defined as sustained ventricular tachycardia or atrial fibrillation with uncontrolled ventricular rate) or cardiomyopathy; poor image quality or missing data; and loss to follow-up.

**Figure 1:**
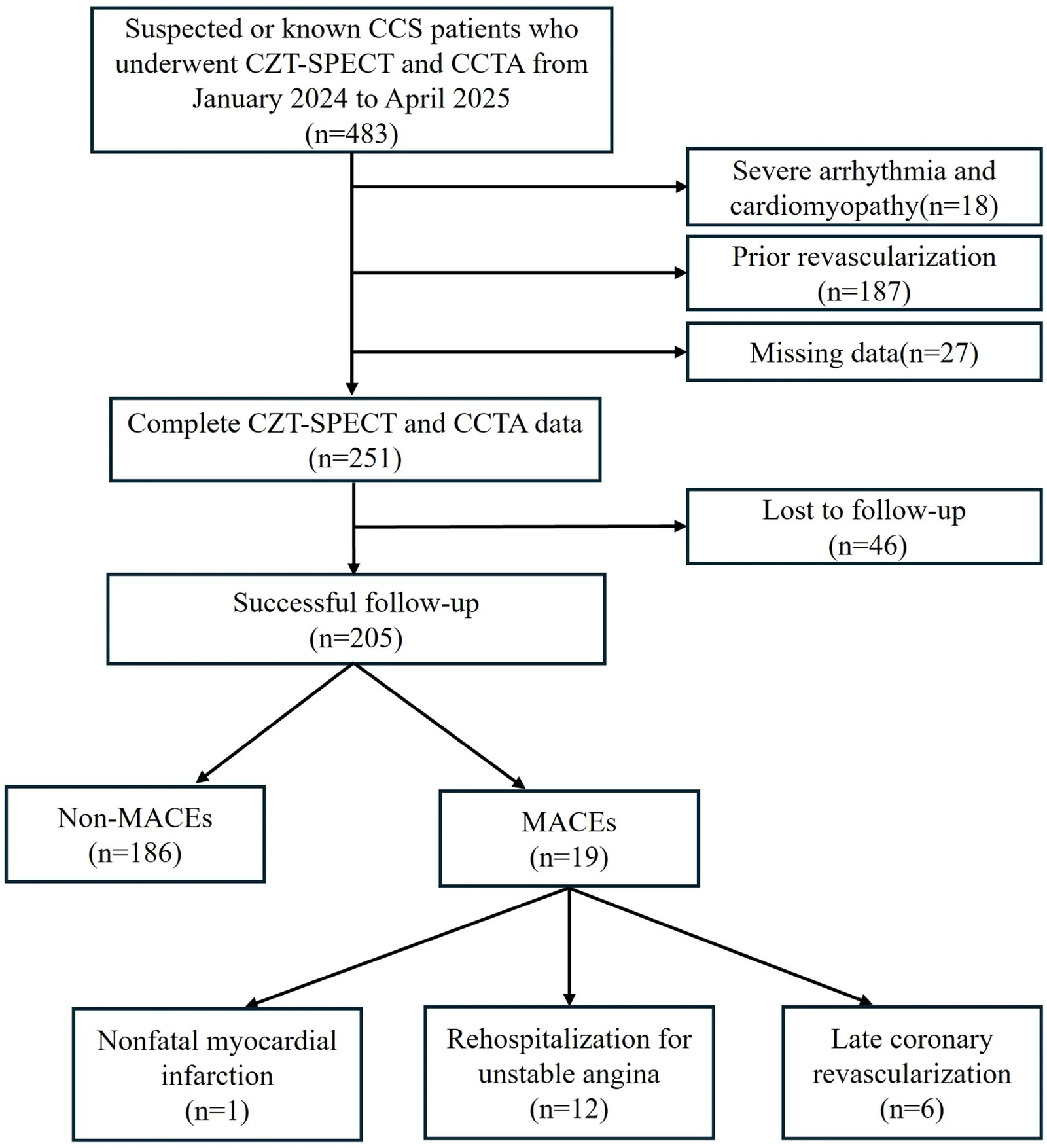
Flowchart of screening and follow-up procedures. CCS: chronic coronary syndromes; CZT-SPECT: cadmium–zinc–telluride single-photon emission computed tomography; CCTA: coronary computed tomography angiography; MACEs: major adverse cardiovascular events.

Short-term follow-up was conducted via telephone or review of electronic medical records to document myocardial ischemic MACEs. The median follow-up time was 19 months (interquartile range (IQR) [16, 23]). Myocardial ischemic MACEs included at least one of the following: non-fatal myocardial infarction, rehospitalization for unstable angina, or late coronary revascularization (percutaneous coronary intervention or coronary artery bypass grafting) [15]. Rehospitalization for unstable angina included: typical anginal symptoms at rest or with minimal exertion, new ECG changes (ST depression ≥ 0.1 mV or T wave inversion), normal cardiac enzymes, and consensus reached by two independent cardiologists after medical record review. The screening and follow-up process is shown in Figure 1. All participants provided written informed consent prior to the examination. The study adhered to the Helsinki Declaration and was approved by our hospital ethics committee (Ethics Number: 2026-0109-3).

### 2.2. Imaging Methods

#### 2.2.1. CZT-SPECT DMPI

CZT-SPECT (NM530c, GE Healthcare, Haifa, Israel) imaging was performed using ^99m^Tc-sestamibi or ^99m^Tc-tetrofosmin. Patients were instructed to abstain from caffeine/theophylline-containing products for 24 h, to withhold cardiovascular medications (e.g., beta-blockers, calcium channel blockers, vasodilators) for 24 h, and to refrain from smoking on the day of the examination [16].

All patients underwent a one-day imaging protocol (rest first, then stress). Pharmacological stress was induced with adenosine (0.14 mg/kg/min for 6 min). At peak stress (3 min), the radiotracer dose was approximately 3–4 times that of the rest dose (185–370 MBq) [17]. Low-dose CT (NM690, GE Healthcare, Milwaukee, WI, USA) was used for attenuation correction. Raw data were transferred to Xeleris 3.0 (GE Healthcare, Milwaukee, WI, USA) and MyoFlowQ 1.0.2 (Beijing Larkcloud Biomedical, Beijing, China) workstations for dynamic SPECT reconstruction and myocardial blood flow quantification. Regions of interest for the input function and left ventricular basal segments were adjusted automatically or manually. Finally, global and vessel-specific stress/rest myocardial blood flow (sMBF/rMBF) and coronary flow reserve (CFR) were calculated.

Gated images were reconstructed using an iterative method, and tomographic images were obtained using quantitative perfusion SPECT (QPS) and quantitative gated SPECT (QGS) software (Version: Cardiac Suite 2013) (Cedars-Sinai Medical Center, Los Angeles, CA, USA). The reconstructed tomographic images included short-axis, vertical long-axis, and horizontal long-axis views of the left ventricle, along with a 17-segment bullseye map according to the American Heart Association (AHA) standards. Semi-quantitative parameters of MPI were calculated using QPS software (Version: Cardiac Suite 2013) [17], including summed stress score (SSS), summed rest score (SRS), and summed difference score (SDS), where SDS = SSS − SRS.

#### 2.2.2. CCTA

Scanning was performed using a GE 256-slice spiral CT scanner (Revolution, GE Healthcare, Chicago, IL, USA) with prospective ECG gating. The contrast agent (Ioversol, 350 mgI/mL, 60–70 mL) was injected at a flow rate of 4.5–5 mL/s. Scanning parameters were 100 or 120 kV and Smart mA (300–740 mA) [18]. The image data were analyzed using AI-assisted software (uAI Discover-CCTA version 1.5, CTA Coronary Intelligence Analysis System, United Imaging Healthcare, Shanghai, China). Plaque segmentation and centerline extraction were manually corrected when necessary.

Statistical parameters included: (a) Coronary artery calcium score (CACS), defined using the Agatston calcification score in Agatston units (AU), with severity levels of 0 (normal), 1–99 (mild), 100–400 (moderate), and >400 (severe). (b) Perivascular fat attenuation index (FAI), defined as the mean CT attenuation (−190 to −30 HU) of perivascular adipose tissue. (c) High-risk plaque features: positive remodeling (remodeling index ≥ 1.1); low-attenuation plaque (mean CT density < 30 HU); napkin-ring sign (ring-like peripheral enhancement around a hypodense core); spotty calcification (<3 mm length and peak density > 130 HU). (d) Plaque burden by type: calcified (>350 HU), lipid (−30 to 30 HU), fibrous fatty (30 to 130 HU), and fibrous plaque (130 to 350 HU). The definitions of the above parameters are provided in [19,20].

### 2.3. Statistical Analysis

Continuous data were first tested for normality. Normally distributed data were expressed as mean ± standard deviation, analyzed using Pearson correlation, and compared between groups using *t*-test or one-way analysis of variance (ANOVA). Non-normally distributed data were expressed as median (IQR), analyzed using Spearman rank correlation, and compared between groups using the Mann–Whitney U test. Categorical data were expressed as count (percentage) and compared using the chi-square test or Fisher’s exact test when appropriate.

Univariate Cox regression was first used to evaluate all variables. Clinically relevant variables or those with univariate association with the outcome were entered into the multivariate Cox regression model. Cumulative incidence of MACEs was estimated using the Kaplan–Meier method and compared by the log-rank test. The combined risk stratification ability of SDS and FAI was evaluated using the area under the curve (AUC) from receiver operating characteristic (ROC) curves, net reclassification improvement (NRI), integrated discrimination improvement (IDI), Kaplan–Meier curves, and the log-rank test. Inverse probability weighting (IPW) sensitivity analysis was used to assess the impact of loss-to-follow-up bias. A sensitivity analysis stratified by the interval between CZT-SPECT and CCTA (≤5 days vs. >5 days) was performed to assess the potential influence of the time interval on the results. Collinearity among covariates was assessed using the variance inflation factor (VIF), with a threshold of VIF < 5 indicating no significant collinearity.

Statistical analysis was performed using SPSS 27.0 (IBM Corp., Armonk, NY, USA) and R software version 4.5.1 (R Foundation for Statistical Computing, Vienna, Austria) with the packages pROC (version 1.19.0.1), PredictABEL (version 1.2-4), nricens (version 1.6), survival (version 3.8-3), and survminer (version 0.5.2). A two-tailed *p*-value < 0.05 was considered statistically significant.

## 3. Results

### 3.1. Clinical Baseline Characteristics

A total of 205 patients with suspected or known CCS who underwent both CZT-SPECT and CCTA were enrolled in this study. The median age was 60 years (IQR [52, 68]), with 89 females (43.4%) and 116 males (56.6%). The follow-up results showed that myocardial ischemic MACEs occurred in 19 patients (9.3%), including non-fatal myocardial infarction in 1 patient (0.5%), rehospitalization for unstable angina in 12 patients (5.9%), and late revascularization in 6 patients (2.9%).

Patients were divided into two groups based on follow-up outcomes: the non-MACEs group and the MACEs group. The clinical baseline characteristics of the two groups are summarized in Table 1. The results showed that the MACEs group had significantly higher rates of hypertension (78.9% vs. 51.6%, *p* = 0.023) and smoking history (52.6% vs. 28.5%, *p* = 0.030) compared with the non-MACEs group. No statistically significant differences were observed in other baseline characteristics (all *p* > 0.05).

### 3.2. Comparison of aCZT-SPECT and CCTA Parameters Between Groups

The comparison of parameters between the two groups showed that among CZT-SPECT parameters, the MACEs group had significantly higher SSS (median 3.51 vs. 2.00, *p* = 0.016) and SDS (median 3.00 vs. 2.00, *p* = 0.010) than the non-MACEs group. Among CCTA coronary parameters, the MACEs group had significantly higher FAI (−75.50 ± 6.99 HU vs. −79.74 ± 6.67 HU, *p* = 0.009), various types of plaque burden (all *p* < 0.05), napkin-ring sign (73.7% vs. 47.8%, *p* = 0.032), and spotty calcification (89.5% vs. 66.1%, *p* = 0.037) compared to the non-MACEs group. No statistically significant differences were observed in other characteristic parameters (all *p* > 0.05), as detailed in Table 2.

### 3.3. Cox Regression Analysis of the Association Between Different Parameters and MACEs

Univariate Cox regression analysis showed that hypertension, smoking history, SDS, FAI, various plaque burdens, and the napkin-ring sign were all significantly associated with MACEs (all *p* < 0.05), as detailed in Table 3.

Three multivariate Cox regression models were further constructed. In Model 1, after adjustment for clinical factors (age and hypertension), SDS showed borderline significance (*p* = 0.062), whereas FAI was significantly associated with MACEs (HR: 1.100, 95%CI: 1.020–1.187, *p* = 0.014). In Model 2, adjusted for other imaging parameters (total plaque burden, napkin-ring sign, and SSS), SDS became a significant predictor (HR: 1.418, 95%CI: 1.094–1.839, *p* = 0.008), while FAI showed borderline significance (*p* = 0.069). In Model 3, adjusted for both clinical factors and imaging parameters, SDS remained an independent predictor of MACEs (HR: 1.386, 95%CI: 1.032–1.861, *p* = 0.030), whereas FAI was no longer significant. See Table 4 for details.

### 3.4. Predictive Value of SDS and FAI for MACE

ROC analysis showed that SDS had a higher AUC (0.678) than FAI (0.659), but the combined model did not improve AUC (0.677). The DeLong test revealed no significant differences among the three (all *p* > 0.05). Based on the Youden index, the cutoff values for SDS and FAI were 1.5 and −78.79 HU, respectively, as detailed in Supplementary Figure S1 and Supplementary Table S1.

**Figure 2:**
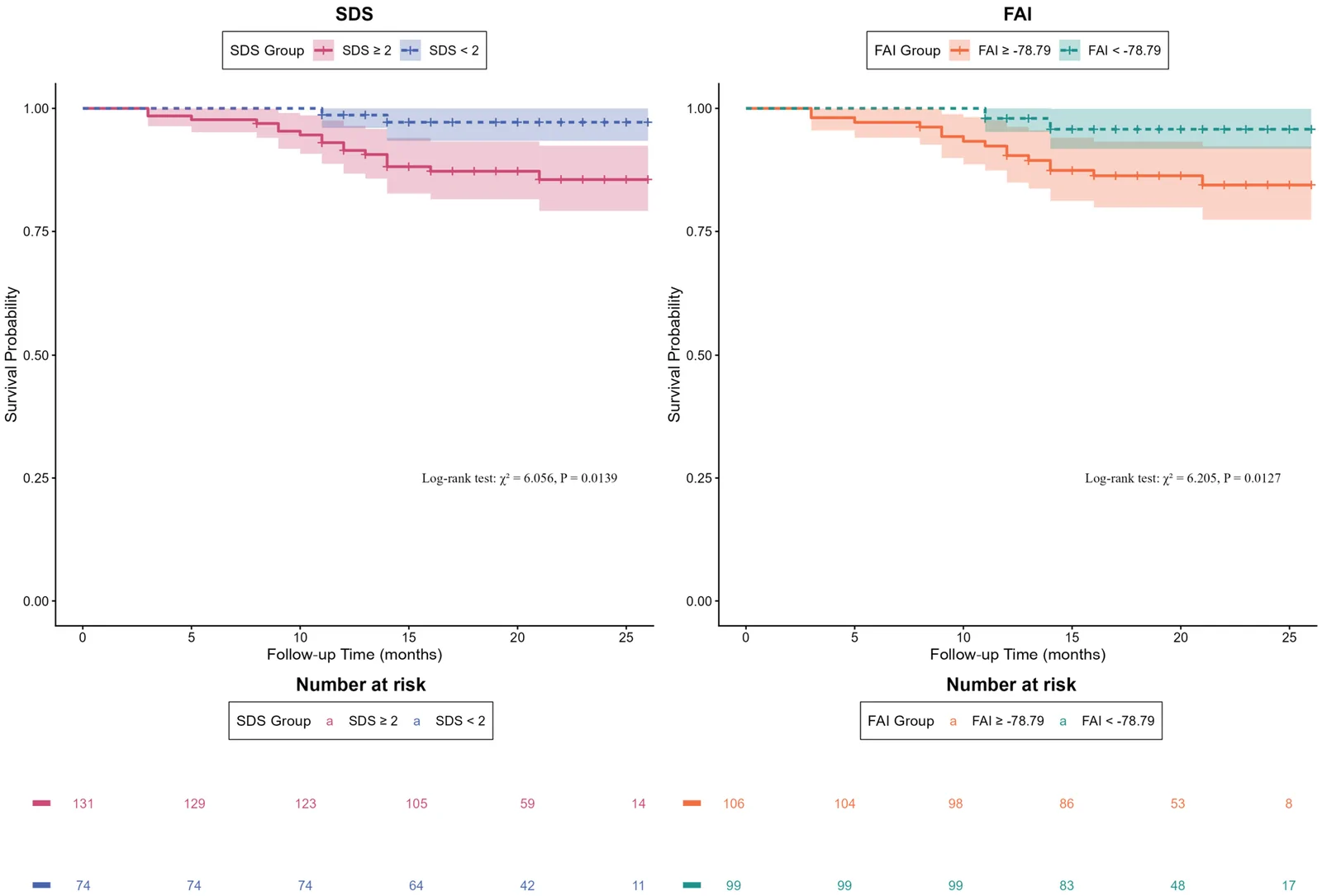
Kaplan–Meier survival curves for SDS and FAI in predicting MACEs. SDS: summed difference score; FAI: perivascular fat attenuation index.

After grouping by cutoff values (an SDS cutoff of 2 was used, as it is an integer score), Kaplan–Meier curves and log-rank tests showed that patients in the high-value groups for both SDS (*p* = 0.014) and FAI (*p* = 0.013) had significantly worse prognosis than those in the low-value groups, as shown in Figure 2.

To evaluate the incremental predictive value of SDS and FAI, four Cox regression models were constructed, and NRI/IDI were calculated. Adding FAI to clinical factors significantly improved risk reclassification (NRI = 0.262, *p* = 0.016) and discrimination (IDI = 0.052, *p* = 0.023), whereas adding SDS did not (all *p* > 0.05). Adding FAI to clinical factors plus SDS still significantly improved prediction (NRI = 0.215, *p* = 0.037; IDI = 0.043, *p* = 0.017), but adding SDS to clinical factors plus FAI did not (all *p* > 0.05), as detailed in Table 5.

**Figure 3:**
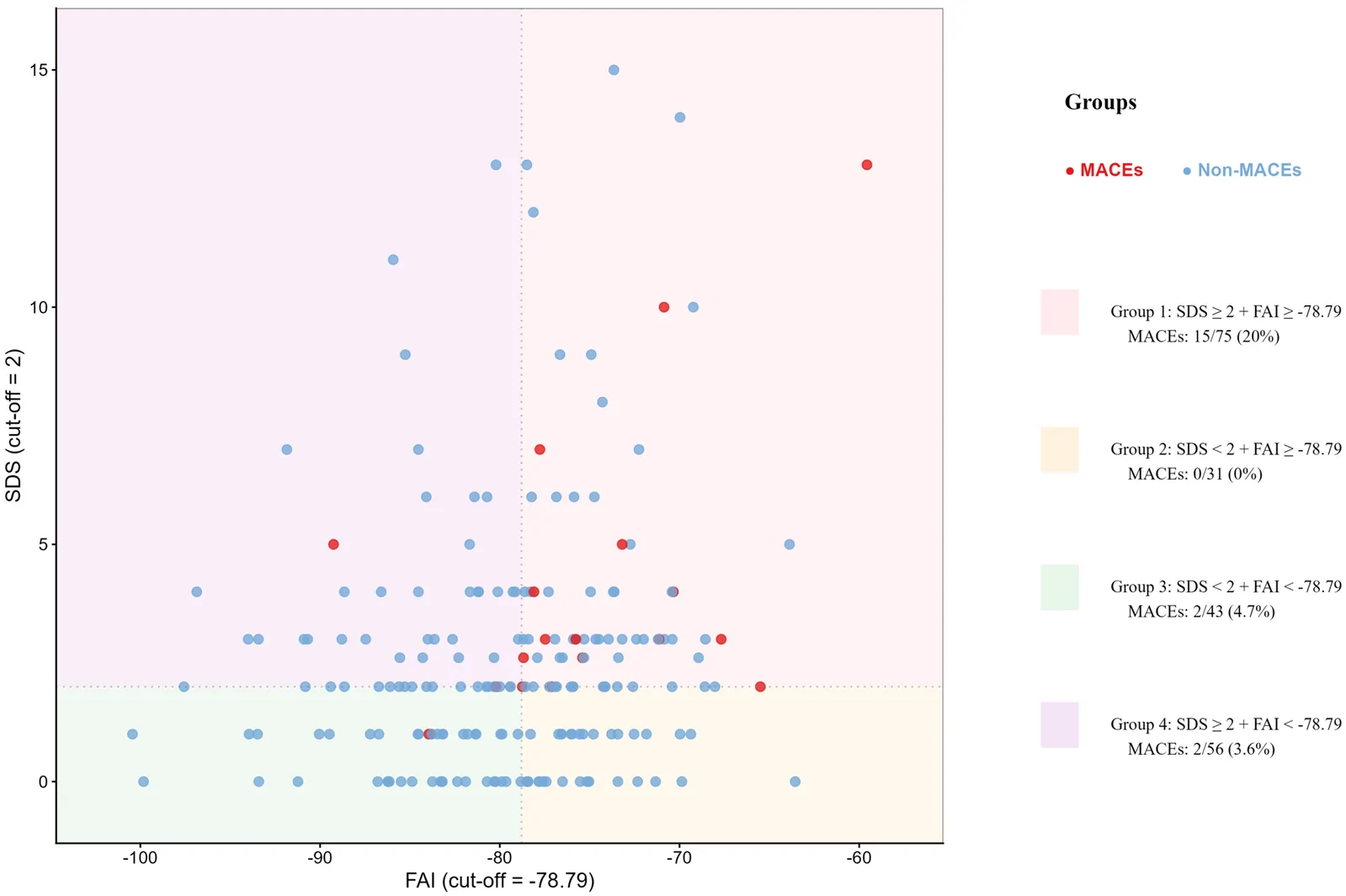
Scatter plot based on the SDS and FAI. SDS: summed difference score; FAI: perivascular fat attenuation index; MACEs: major adverse cardiovascular events.

**Figure 4:**
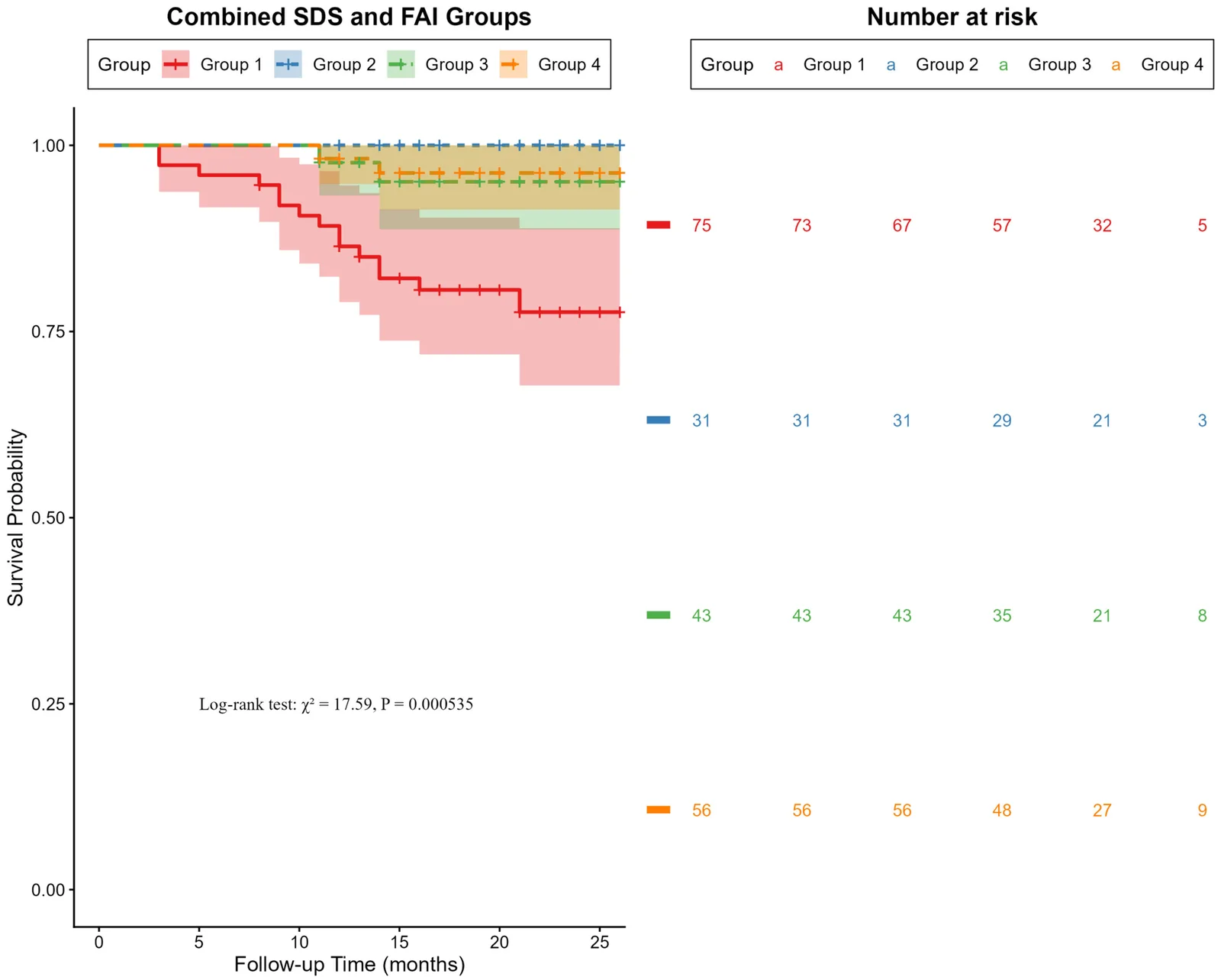
Kaplan–Meier survival curves for combined SDS and FAI stratification in predicting MACEs. Group 1: SDS ≥ 2 + FAI ≥ −78.79; Group 2: SDS < 2 + FAI ≥ −78.79; Group 3: SDS < 2 + FAI < −78.79; Group 4: SDS ≥ 2 + FAI < −78.79. SDS: summed difference score; FAI: perivascular fat attenuation index.

Based on the cutoff values, SDS and FAI were combined into four strata. The corresponding Kaplan–Meier survival curves and log-rank tests showed that the event rate in Group 1 (SDS ≥ 2 + FAI ≥ −78.79) was as high as 20.0%, which was significantly higher than that in the other groups (all *p* < 0.05), as detailed in Figure 3 and Figure 4 and Supplementary Tables S2 and S3.

### 3.5. Correlation Analysis Between SDS and CFR

Spearman correlation analysis revealed a weak negative correlation between the two parameters (r = −0.215, *p* = 0.002), as shown in Supplementary Figure S2.

### 3.6. Assessment of Loss-to-Follow-Up Bias

A comparison between the lost-to-follow-up group and the follow-up group showed that the lost-to-follow-up group had significantly lower rates of family history (2.2% vs. 16.1%, *p* = 0.013) and lower CFR (median 1.91 vs. 2.42, *p* = 0.034). No significant differences were observed for other variables (all *p* > 0.05). See Supplementary Table S4 for details.

IPW sensitivity analysis showed that SDS was not significant (HR = 1.102, *p* = 0.099), whereas FAI was significantly associated with MACEs (HR = 1.100, *p* = 0.015) in the unweighted Cox regression analysis. After IPW adjustment, both weighted models demonstrated that SDS (HR: 1.103–1.110, *p* < 0.05) and FAI (HR: 1.094–1.096, *p* < 0.05) were significantly associated with MACEs. See Supplementary Table S5 for details.

### 3.7. Sensitivity Analysis Stratified by Examination Interval

The median interval between CZT-SPECT and CCTA was 3 days (IQR: 1–5 days), with 97.6% within 30 days. The sensitivity analysis showed no significant differences in core imaging parameters (SDS, FAI, plaque burden) or MACE (all *p* > 0.05), except for age (*p* = 0.014). See Supplementary Table S6 for details.

**Figure 5:**
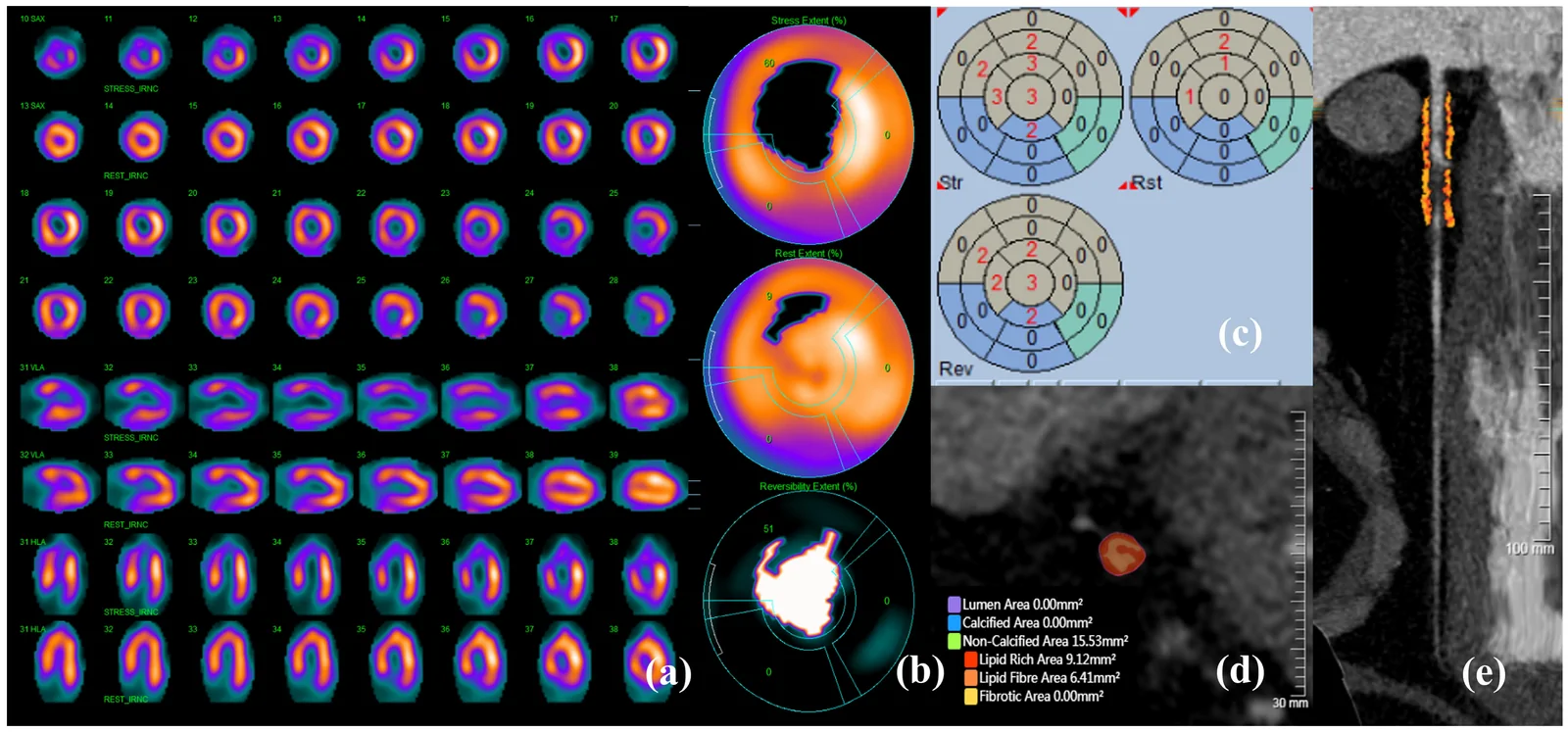
A typical case example. A 37-year-old male patient was admitted with intermittent precordial pain lasting six months. (**a**) Myocardial perfusion tomographic images (odd rows: stress images; even rows: rest images; rows 1–4: short-axis images; rows 5–6: vertical long-axis images; rows 7–8: horizontal long-axis images). (**b**) Myocardial perfusion polar map showing extensive reversible changes in radioactive distribution involving the left ventricular apex, part of the anterior wall, and anteroseptal wall (accounting for approximately 25% of the left ventricular wall area), along with a small area of fixed sparse radioactivity in the mid-anterior wall (accounting for approximately 5% of the left ventricular wall area). (**c**) QPS/QGS quantitative analysis results: SSS = 15, SRS = 4, SDS = 11. (**d**) AI-based quantitative analysis of the plaque in the mid-segment of the LAD, showing that the plaque is a non-calcified plaque with two high-risk plaque features: positive remodeling and low-attenuation plaque. (**e**) AI-automated measurement of FAI around the LAD: −87.13 HU.

A typical case is shown in Figure 5.

## 4. Discussion

This study suggests that SDS may serve as an independent predictor of short-term ischemic MACEs in CCS patients, while FAI may offer incremental predictive value and enable finer risk stratification in patients with elevated SDS. Patients with both high SDS and high FAI appear to be at the highest risk in this cohort. Notably, the predictive values of SDS and FAI remained consistent before and after adjusting for loss-to-follow-up bias, supporting the robustness of our findings. Of particular note, the relatively short follow-up period (median 19 months) in this study allowed us to focus primarily on short-term risk prediction. Moreover, all MACEs were ischemia-related events, which avoids confounding from non-ischemic events such as heart failure and stroke. For example, myocardial infarction indicated acute myocardial injury with concurrent acute ischemia [21], whereas unstable angina and late revascularization were driven by symptoms or progression of myocardial ischemia.

Kaplan–Meier analysis showed that the high-SDS group had a significantly lower event-free survival rate, indicating good univariable stratification ability of SDS. In multivariate Cox regression, SDS was borderline significant after adjusting for clinical factors, but remained significant after adjusting for other imaging parameters. IPW sensitivity analysis confirmed the robustness of SDS. After adjusting for loss-to-follow-up probability, the HR direction remained consistent with the primary analysis, and significance increased from *p* = 0.099 to *p* < 0.05, suggesting that loss-to-follow-up bias may have underestimated its predictive value. These findings indicate that SDS, as a semi-quantitative measure of reversible myocardial ischemia burden, provides independent predictive value and serves as a stable predictor of short-term ischemic events in CCS patients. Conventional SPECT MPI is the most widely used technique for assessing CAD. It detects reduced tracer uptake in myocardial regions relative to normal areas, reflecting relative myocardial perfusion, to diagnose ischemia and stratify risk [22]. Borren et al. showed [23] that semi-quantitative assessment (SDS ≥ 2 and SSS ≥ 4) detected ischemia more frequently than visual assessment, and SDS < 4 with low/intermediate pretest probability effectively ruled out ischemia. A long-term study of 717 women with abnormal SDS (≥1) found [24] that different SDS strata corresponded to different MACE prediction models, indicating that SDS is a continuous risk marker with hierarchical predictive value; as SDS severity increased, the prediction models simplified, suggesting SDS stratification enables more accurate and streamlined risk assessment.

Meanwhile, patients in the high FAI group had significantly lower event-free survival than the low FAI group (log-rank *p* = 0.013). Univariate Cox regression confirmed that FAI was significantly associated with MACEs, indicating that FAI also has good univariate predictive ability. However, in multivariate Cox regression, FAI remained significant after adjustment for clinical factors but lost significance after further adjustment for other imaging parameters or after full adjustment. This result suggests that the predictive information of FAI may overlap with that of other imaging parameters (such as plaque burden and SSS). As a marker of perivascular inflammation, FAI reflects endothelial dysfunction and subsequent “functional stenosis” [25]. Thus, its independent contribution is attenuated when these parameters are included together in the model. Previous studies have confirmed [26,27,28] that as a quantitative marker of coronary inflammation, FAI not only predicts CAD prognosis and improves risk stratification but also guides early targeted prevention. A recent study of 503 young patients with suspected CAD showed [29] that over a median follow-up of 72.7 months, the high FAI group (FAI ≥ −75.2 HU) had a significantly higher risk of MACEs (HR = 19.257, *p* = 0.004), demonstrating potential risk stratification value in this population. Moreover, studies have shown that in patients after coronary artery bypass grafting, FAI is an independent predictor of graft occlusion (HR = 5.205, *p* = 0.001) [30]; in patients after stent implantation, FAI independently predicts in-stent restenosis (OR = 1.403, *p* < 0.001) [31].

NRI and IDI analyses showed that adding FAI to clinical factors significantly improved risk prediction, whereas adding SDS did not; adding FAI to clinical factors plus SDS also significantly improved risk reclassification, while adding SDS to clinical factors plus FAI did not. This indicates that, even though FAI’s effect was partially absorbed by correlated covariates in the multivariable Cox model, it still provides incremental predictive information beyond SDS. Biologically, FAI represents an upstream inflammatory signal, while SDS reflects a downstream ischemic consequence—they are related but not completely overlapping. Notably, in patients with high SDS, FAI further identifies those with superimposed inflammatory activity. The four-group Kaplan–Meier curves further support this: only patients with both high SDS and high FAI were at the highest risk (event rate 20.0%), whereas those with high SDS but low FAI had a risk comparable to those with low SDS. Although SDS was independently significant in multivariate Cox regression, its NRI and IDI based on clinical factors were not significant, likely because clinical factors (age, hypertension) already explained most risk variation, and the limited number of MACEs reduced the statistical power to detect the modest incremental effect of SDS. Nevertheless, the independent predictive value of SDS was supported by the multivariate analysis, while the NRI and IDI results further suggested the complementary value of FAI beyond SDS. This is consistent with previous studies. Zheng et al. showed [32] that FAI provided incremental predictive value beyond clinical variables and CCTA features in 1614 patients with non-obstructive CAD. Similarly, another study demonstrated [33] that FAI added incremental prognostic value regarding CAD extent and severity beyond conventional CCTA parameters.

This study found that while combining SDS and FAI did not significantly improve overall predictive performance (AUC = 0.677, all *p* > 0.05), this does not represent a contradiction. Cox regression addresses independent prognostic value, NRI/IDI evaluate improvement in individual risk classification and integrated discrimination, and ROC assesses overall discrimination—these methods are complementary rather than conflicting. Together, they indicate that FAI’s value lies not in improving overall AUC, but in refining risk stratification within the SDS-high subgroup. Thus, these findings raise the hypothesis that a two-step risk assessment strategy—SDS-based screening followed by FAI assessment in high-SDS patients—might help identify individuals at particularly high risk (high SDS + high FAI). This hypothesis warrants prospective validation before clinical application.

Notably, in the four-group analysis, Group 2 (SDS < 2 + FAI ≥ −78.79) had an event rate of 0.0%. This may be explained by the small sample size (31 cases) potentially leading to sampling error, the relatively short median follow-up of 19 months, and the pathophysiological state of “active inflammation without ischemia”, which may indeed confer low short-term risk. On the other hand, the quantitative parameters CFR and MBF showed no significant predictive value, whereas the semi-quantitative parameter SDS effectively distinguished MACE risk. Spearman correlation analysis revealed a weak negative correlation between CFR and SDS (r = −0.215, *p* = 0.002). We believe this indicates that the two parameters measure related but different biological dimensions. It is well known that CFR reflects global coronary flow reserve (including epicardial vessels and microcirculation) [34], whereas SDS is a semi-quantitative 17-segment model-based score that captures regional segmental ischemia [22], potentially being more sensitive to plaque-driven ischemic events. Moreover, possible explanations include the limited number of events (19 cases), which may be insufficient to detect a weak predictive signal from CFR, and the relatively small variability of CFR in a stable population, further limiting its discriminative ability. In addition, the sensitivity analysis stratified by the examination interval showed that the two groups were largely comparable. The minor age difference does not appear to have influenced the main conclusions, as age was included as a covariate in all multivariable models, and the core imaging parameters as well as MACEs were balanced between groups. Taken together, these findings suggest that the time interval between the two examinations does not materially affect the primary results.

In a recent study, Fogante et al. demonstrated [35] the feasibility of a combined CCTA and stress dynamic CT myocardial perfusion imaging protocol for integrated anatomical and functional assessment of moderate CAD, which supports the value of multiparametric non-invasive imaging in CAD. In line with this concept, our study extends the multiparametric approach to prognosis, showing that a cross-modality combination of CZT-SPECT (perfusion) and CCTA (inflammation) identifies a high-risk CCS subgroup, further reinforcing the role of multimodality imaging in CCS management. Additionally, the 2024 ESC guidelines [3] explicitly acknowledge that combined anatomical and functional imaging (e.g., CCTA with PET) may improve prognostication in CCS patients, and that sequential testing may be needed in intermediate-likelihood populations to avoid false positives. Our findings align with this recommendation by providing prognostic evidence that adding CCTA-derived FAI to CZT-SPECT-derived SDS further identifies a high-risk subgroup (event rate 20.0%) among CCS patients. This supports the potential role of combined imaging beyond diagnostic evaluation, extending to risk stratification and therapeutic decision-making.

Future studies may further explore the role of emerging imaging techniques in enhancing the prognostic framework for CCS patients. Cardiac magnetic resonance (CMR) [36] allows for assessment of the non-infarcted myocardium after acute myocardial infarction, providing prognostic information beyond traditional parameters. Photon-counting CT (PCCT) [37], with its improved spatial and contrast resolution and multi-energy quantitative imaging capabilities, holds promise for more precise characterization of coronary plaques and myocardial tissues. Integrating these advanced tools into the multiparametric prognostic framework may further improve risk stratification in CCS patients.

This study has several limitations. First, it was a single-center, retrospective study, which may introduce selection bias. Second, the limited number of MACEs (n = 19) may have reduced statistical power and increased the risk of overfitting in the multivariable analyses. Although we restricted the number of covariates in each model and confirmed that all VIF values were < 5 to minimize this risk, some degree of residual overfitting cannot be excluded. Furthermore, this restriction also meant that some parameters—such as plaque burden subtypes—although associated with MACEs in univariable analyses, could not be included due to collinearity or event number constraints; their additional predictive value warrants further evaluation in larger cohorts. Accordingly, the present findings should be interpreted as exploratory and hypothesis-generating, requiring confirmation in prospective studies with larger sample sizes. Third, the loss to follow-up rate was 18.3% (46/251); although IPW sensitivity analysis suggested limited impact, unmeasured confounding may still exist. Fourth, the FAI cutoff value (−78.79 HU) was determined based on ROC analysis in this cohort and requires further validation for generalizability.

## 5. Conclusions

This study suggests that SDS may independently predict short-term myocardial ischemic events, and that FAI may provide incremental predictive value. Patients with both high SDS and high FAI are the highest-risk population, and their combination is valuable for clinical risk stratification. A two-step approach may be considered as a hypothesis-generating strategy: first, SDS from CZT-SPECT MPI to screen for reversible ischemia; second, FAI from CCTA for refined risk stratification. Leveraging multi-parameter information from both modalities may improve risk stratification accuracy in CCS patients. However, these findings require validation in multicenter studies with larger samples and longer follow-up.

## Figures and Tables

**Table 1:** Baseline clinical characteristics:

Variable	Non-MACEs (n = 186)	MACEs (n = 19)	*p* Value
Age, years	60.00 (52.75, 68.00)	58.00 (45.00, 70.00)	0.463
Man, n (%)	102 (54.8%)	14 (73.7%)	0.114
BMI, kg/m^2^	25.12 (23.31, 28.38)	25.92 (22.47, 27.77)	0.842
Hypertension, n (%)	96 (51.6%)	15 (78.9%)	0.023
Diabetes, n (%)	35 (18.8%)	3 (15.8%)	0.746
Hyperlipidemia, n (%)	51 (27.4%)	6 (31.6%)	0.700
Smoking history, n (%)	53 (28.5%)	10 (52.6%)	0.030
Family history, n (%)	32 (17.2%)	1 (5.3%)	0.177

BMI: Body mass index.

**Table 2:** Comparison of CZT-SPECT and CCTA parameters between the groups.

Variable	Non-MACEs (n = 186)	MACEs (n = 19)	*p* Value
CFR	2.48 ± 0.88	2.41 ± 1.11	0.756
rMBF	0.80 (0.75, 0.84)	0.78 (0.71, 0.85)	0.736
sMBF	1.91 (1.32, 2.51)	1.59 (1.18, 2.38)	0.356
SSS	2.00 (1.00, 4.00)	3.51 (3.00, 6.00)	0.016
SRS	0.00 (0.00, 1.00)	0.00 (0.00, 1.00)	0.369
SDS	2.00 (1.00, 3.00)	3.00 (2.00, 5.00)	0.010
CACS (AU)	581.88 (372.32, 807.88)	401.30 (268.66, 715.19)	0.174
FAI (HU)	−79.74 ± 6.67	−75.50 ± 6.99	0.009
Total Plaque Burden (%)	24.18 (7.42, 47.79)	76.82 (34.55, 114.11)	<0.001
Calcified Plaque Burden (%)	2.82 (0.45, 11.37)	7.78 (6.11, 25.80)	0.002
Non-Calcified Burden (%)	18.86 (6.42, 37.26)	48.74 (19.43, 73.19)	<0.001
Lipid Plaque Burden (%)	0.96 (0.17, 2.67)	2.76 (0.79, 5.49)	0.012
Fibrous Fatty Burden (%)	6.12 (1.88, 13.25)	17.82 (4.47, 24.54)	0.002
Fibrotic Plaque Burden (%)	9.85 (3.58, 20.85)	25.37 (12.78, 43.54)	<0.001
Positive Remodeling	160 (86.0%)	18 (94.7%)	0.479
Low-Attenuation Plaque	162 (87.1%)	18 (94.7%)	0.479
Napkin-ring Sign	89 (47.8%)	14 (73.7%)	0.032
Spotty Calcification	123 (66.1%)	17 (89.5%)	0.037

CFR: coronary flow reserve; rMBF: rest myocardial blood flow; sMBF: stress myocardial blood flow; SSS: summed stress score; SRS: summed rest score; SDS: summed difference score; CACS: coronary artery calcium score; FAI: perivascular fat attenuation index.

**Table 3:** Univariate Cox regression analysis: association between various parameters and MACEs.

Variable	HR	95%CI	*p* Value
Age, years	0.981	0.940–1.023	0.370
Man, n (%)	2.253	0.811–6.254	0.119
BMI, kg/m^2^	1.006	0.888–1.140	0.921
Hypertension, n (%)	3.429	1.138–10.335	0.029
Diabetes, n (%)	0.801	0.233–2.750	0.724
Hyperlipidemia, n (%)	1.225	0.466–3.224	0.681
Smoking history, n (%)	2.795	1.135–6.881	0.025
Family history, n (%)	0.296	0.040–2.220	0.236
CFR	0.916	0.546–1.538	0.741
rMBF	0.025	0.000–3.668	0.147
sMBF	0.790	0.426–1.466	0.455
SSS	1.044	0.970–1.123	0.253
SRS	0.955	0.746–1.222	0.714
SDS	1.131	1.009–1.268	0.035
CACS (AU)	0.999	0.998–1.001	0.448
FAI (HU)	1.109	1.028–1.197	0.008
Total Plaque Burden (%)	1.008	1.004–1.012	<0.001
Calcified Plaque Burden (%)	1.012	1.003–1.021	0.011
Non-Calcified Burden (%)	1.019	1.010–1.028	<0.001
Lipid Plaque Burden (%)	1.132	1.044–1.227	0.003
Fibrous Fatty Burden (%)	1.057	1.029–1.085	<0.001
Fibrotic Plaque Burden (%)	1.029	1.015–1.043	<0.001
Positive Remodeling	2.840	0.379–21.278	0.310
Low-Attenuation Plaque	2.588	0.345–19.387	0.355
Napkin-ring Sign	3.028	1.091–8.410	0.033
Spotty Calcification	4.154	0.960–17.979	0.057

BMI: body mass index; CFR: coronary flow reserve; rMBF: rest myocardial blood flow; sMBF: stress myocardial blood flow; SSS: summed stress score; SRS: summed rest score; SDS: summed difference score; CACS: coronary artery calcium score; FAI: perivascular fat attenuation index.

**Table 4:** Multivariate Cox regression analysis: association between SDS and FAI of MACEs.

Variable	Adjust for Clinical Factors		Adjust for Imaging Indicators		Adjust for Combined	
	HR (95%CI)	*p* Value	HR (95%CI)	*p* Value	HR (95%CI)	*p* Value
SDS	1.114 (0.994–1.247)	0.062	1.418 (1.094–1.839)	0.008	1.386 (1.032–1.861)	0.030
FAI	1.100 (1.020–1.187)	0.014	1.079 (0.994–1.172)	0.069	1.059 (0.977–1.149)	0.163

Clinical factors include age and hypertension; imaging indicators include total plaque burden, napkin-ring sign, and SSS. SDS: summed difference score; FAI: perivascular fat attenuation index; SSS: summed stress score.

**Table 5:** NRI and IDI analyses: incremental predictive value of SDS and FAI.

Basic Model	New Model	NRI		IDI	
		NRI (95%CI)	*p* Value	IDI (95%CI)	*p* Value
clinical factors	+SDS	0.136 (−0.030–0.302)	0.107	0.023 (−0.009–0.055)	0.163
clinical factors	+FAI	0.262 (0.050–0.475)	0.016	0.052 (0.007–0.097)	0.023
clinical factors + FAI	+SDS	0.100 (−0.042–0.242)	0.167	0.013 (−0.010–0.037)	0.257
clinical factors + SDS	+FAI	0.215 (0.013–0.416)	0.037	0.043 (0.007–0.078)	0.017

Clinical factors include age and hypertension. NRI: net reclassification improvement; IDI: integrated discrimination improvement; SDS: summed difference score; FAI: perivascular fat attenuation index.

## Data Availability

The data presented in this study are available on request from the corresponding author due to ethical restrictions and patient privacy protection.

## Supplementary Materials

The following supporting information can be downloaded at: https://www.mdpi.com/article/10.3390/jcdd13090421/s1, Figure S1: ROC curves of SDS, FAI, and their combination for predicting MACEs; Figure S2: Correlation Analysis between CFR and SDS; Table S1: ROC curve analysis and DeLong test of SDS, FAI, and their combination for predicting MACEs; Table S2: Distribution of MACEs across groups after combined SDS and FAI stratification; Table S3: Log-rank test for combined SDS and FAI stratification; Table S4: Comparison between the lost-to-follow-up group and the follow-up group; Table S5: IPW sensitivity analysis for loss to follow-up bias; Table S6: Sensitivity analysis stratified by the examination interval.

## Abbreviations

The following abbreviations are used in this manuscript.

CVD	cardiovascular disease
IHD	ischemic heart disease
DALYs	disability-adjusted life years
CAD	coronary artery disease
ACS	acute coronary syndromes
CCS	chronic coronary syndromes
MACEs	major adverse cardiovascular events
SPECT	single-photon emission computed tomography
MPI	myocardial perfusion imaging
CZT	cadmium–zinc–telluride
CCTA	coronary computed tomography angiography
AI	artificial intelligence
IQR	interquartile range
sMBF	stress myocardial blood flow
rMBF	rest myocardial blood flow
CFR	coronary flow reserve
QPS	quantitative perfusion SPECT
QGS	quantitative gated SPECT
AHA	American Heart Association
SSS	summed stress score
SRS	summed rest score
SDS	summed difference score
CACS	coronary artery calcium scores
FAI	perivascular fat attenuation index
AUC	area under the curve
ROC	receiver operating characteristic
NRI	net reclassification improvement
IDI	integrated discrimination improvement
IPW	inverse probability weighting
VIF	variance inflation factor
CMR	cardiac magnetic resonance
PCCT	Photon-counting CT
