# Supplementary material for: Value of Multi-Parameter Combined Application of CZT-SPECT and CCTA in Risk Stratification of Short-Term Myocardial Ischemic Events in Patients with Chronic Coronary Syndromes
Source: J Cardiovasc Dev Dis. 2026 Aug 28;13(9):421. doi: 10.3390/jcdd13090421 (PMC13607398; doi:10.3390/jcdd13090421)
Supplement: Supplementary file 1 [file jcdd-13-00421-s001.zip › jcdd-4405830-supplementary.pdf]

Supplementary Material

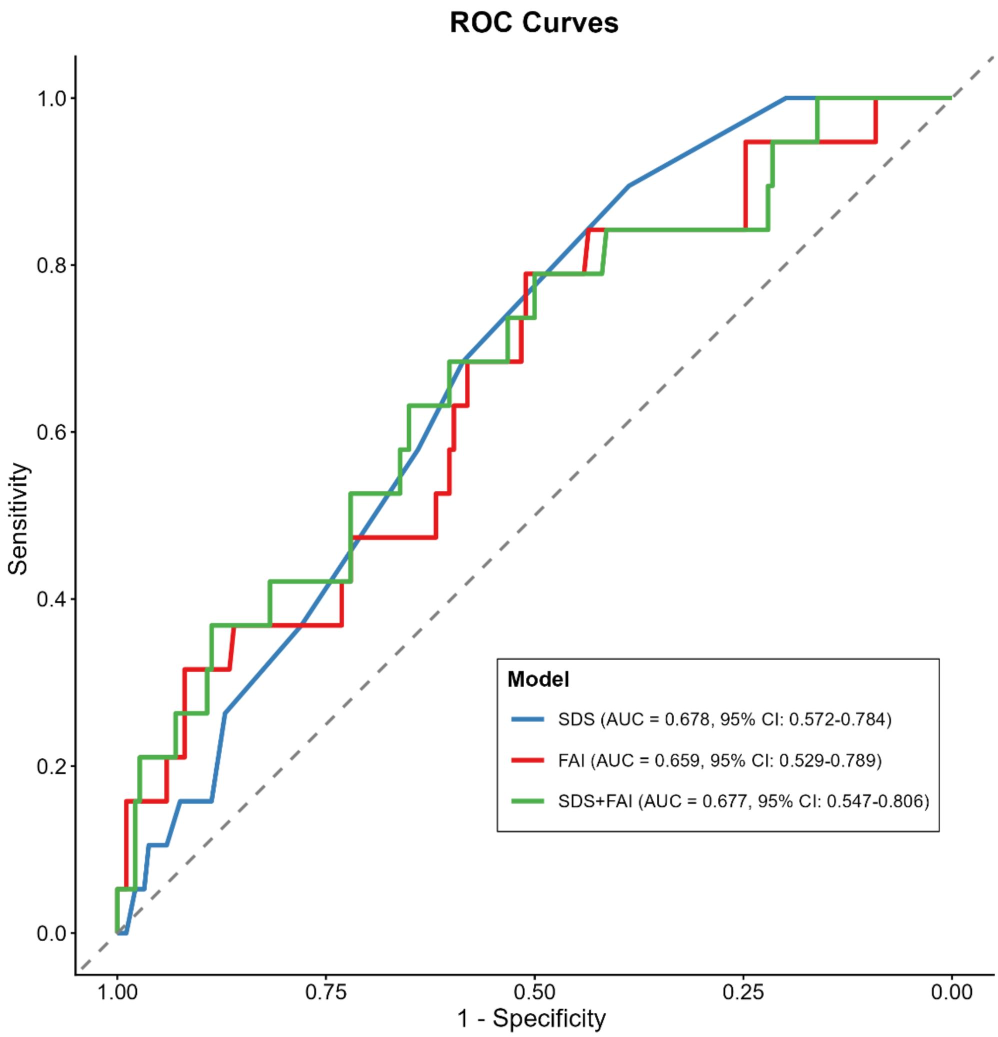

Supplementary Figure S1. ROC curves of SDS, FAI, and their combination for predicting MACEs

SDS: summed difference score; FAI: perivascular fat attenuation index.

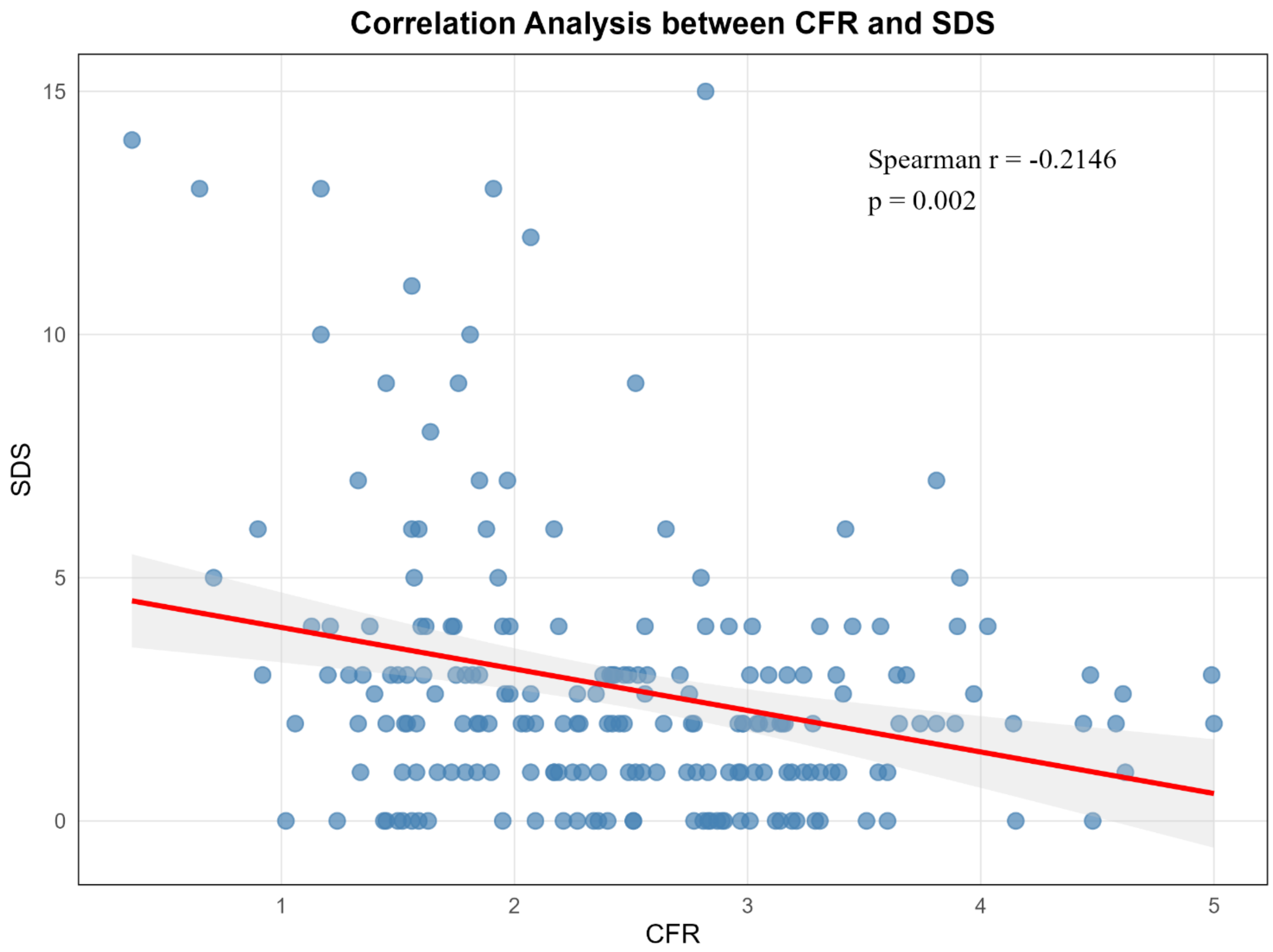

Supplementary Figure S2. Correlation Analysis between CFR and SDS

SDS: summed difference score; FAI: perivascular fat attenuation index.

Supplementary Table S1. ROC curve analysis and DeLong test of SDS, FAI, and their combination for predicting MACEs

Model	AUC	95%CI	Sensitivity	Specificity	Cut-off	P value for DeLong Test
SDS	0.678	0.572-0.784	89.5%	38.7%	1.5	0.978
FAI	0.659	0.529-0.789	78.9%	51.1%	-78.79	0.282
SDS+FAI	0.677	0.547-0.806	78.9%	50.0%	-	-

P value for DeLong Test about FAI vs. SDS is 0.772.

SDS: summed difference score; FAI: perivascular fat attenuation index.

Supplementary Table S2. Distribution of MACEs across groups after combined SDS and FAI stratification

Groups	Total Quantity	MACEs Quantity	Proportion
Group1: SDS ≥2 + FAI ≥-78.79	75	15	20.0%
Group2: SDS <2 + FAI ≥-78.79	31	0	0.0%
Group3: SDS <2 + FAI <-78.79	43	2	4.7%
Group4: SDS ≥2 + FAI <-78.79	56	2	3.6%

SDS: summed difference score; FAI: perivascular fat attenuation index; MACEs: major adverse cardiovascular events.

Supplementary Table S3. Log-rank test for combined SDS and FAI stratification

	χ^2	Z value	P value
Group1 vs. Group2	7.231	2.689	0.007
Group1 vs. Group3	5.188	2.278	0.023
Group1 vs. Group4	7.681	2.771	0.006
Group2 vs. Group3	1.510	-1.229	0.219
Group2 vs. Group4	1.142	-1.069	0.285
Group3 vs. Group4	0.079	-0.281	0.778

Group1: SDS ≥2 + FAI ≥-78.79; Group2: SDS <2 + FAI ≥-78.79; Group3: SDS <2 + FAI <-78.79; Group4: SDS ≥2 + FAI <-78.79.

SDS: summed difference score; FAI: perivascular fat attenuation index.

Supplementary Table S4. Comparison between the lost-to-follow-up group and the follow-up group

Variables	Lost group(n=46)	Follow-up group(n=205)	P value
Age, years	58.50(46.75, 68.00)	60.00(52.00, 68.00)	0.475
Man, n (%)	28(60.9%)	116(56.6%)	0.595
BMI, kg/m2	25.63(23.80, 26.95)	25.22(23.23, 30.39)	0.864
Hypertension, n (%)	24(52.2%)	111(54.1%)	0.808
Diabetes, n (%)	9(19.6%)	38(18.5%)	0.872
Hyperlipidemia, n (%)	13(28.3%)	57(27.8%)	0.950
Smoking history, n (%)	17(37.0%)	63(30.7%)	0.413
Family history, n (%)	1(2.2%)	33(16.1%)	0.013
CFR	1.91(2.01, 3.48)	2.42(1.76, 3.09)	0.034
rMBF	0.80(0.75, 0.84)	0.80(0.75, 0.84)	0.979
sMBF	2.32(1.42, 2.80)	1.89(1.32, 2.50)	0.092
SSS	3.00(1.00, 3.63)	3.00(1.00, 4.00)	0.601
SRS	0.00(0.00, 0.84)	0.00(0.00, 1.00)	0.560
SDS	2.00(1.00, 3.00)	2.00(1.00, 3.00)	0.489
CACS (AU)	547.23(303.05, 760.50)	564.47(342.63, 806.14)	0.213
FAI (HU)	-78.18(-82.45, -74.99)	-78.61(-83.75, -74.93)	0.764
Total Plaque Burden (%)	21.75(8.35, 47.50)	27.22(8.51, 53.72)	0.221
Calcified Plaque Burden (%)	2.29(0.09, 12.64)	3.37(0.47, 12.92)	0.171
Non-Calcified Burden (%)	17.64(5.67, 30.67)	20.52(6.96, 40.68)	0.208

Lipid Plaque Burden (%)	0.99(0.15, 2.22)	1.05(0.22, 2.93)	0.359
Fibrous Fatty Burden (%)	6.62(1.61,11.53)	6.28(2.18, 14.53)	0.341
Fibrotic Plaque Burden (%)	7.51(3.54, 18.42)	11.53(3.69, 23.28)	0.156
Positive Remodeling	38(82.6%)	178(86.8%)	0.455
Low Attenuation Plaque	39(84.8%)	180(87.8%)	0.579
Napkin-ring Sign	19(41.3%)	103(50.2%)	0.273
Spotty Calcification	25(54.3%)	140(68.3%)	0.072

BMI: body mass index; CFR: coronary flow reserve; rMBF: rest myocardial blood flow; sMBF: stress myocardial blood flow; SSS: summed stress score; SRS: summed rest score; SDS: summed difference score; CACS: coronary artery calcium scores; FAI: perivascular fat attenuation index.

Supplementary Table S5. IPW sensitivity analysis for loss to follow-up bias

Method	SDS		FAI	
	HR (95%CI)	P value	HR (95%CI)	P value
Unweighted	1.102(0.982-1.236)	0.099	1.100(1.019-1.188)	0.015
IPW Model 1	1.103(1.010-1.204)	0.030	1.096(1.014-1.184)	0.020
IPW Model 2	1.110(1.017-1.212)	0.020	1.094(1.013-1.182)	0.022

Model 1: Propensity score model adjusted for family history and CFR.

Model 2: Propensity score model adjusted for family history, CFR, SDS, and FAI.

IPW: inverse probability weighting; SDS: summed difference score; FAI: perivascular fat attenuation index.

Supplementary Table S6. Sensitivity analysis stratified by the examination interval

Variables	≤5 day(n=160)	>5 day(n=45)	P value
Age, years	60.50(53.00, 68.75)	55.00(45.50, 63.50)	0.014
Man, n (%)	90(56.3%)	26(57.8%)	0.855
BMI, kg/m2	25.39 ± 3.51	26.55 ± 3.61	0.053
Hypertension, n (%)	86(53.8%)	25(55.6%)	0.830
Diabetes, n (%)	28(17.5%)	10(22.2%)	0.471
Hyperlipidemia, n (%)	42(26.3%)	15(33.3%)	0.349
Smoking history, n (%)	48(30.0%)	15(33.3%)	0.669
Family history, n (%)	24(15.0%)	9(20.0%)	0.420
CFR	2.41(1.77, 3.07)	2.49(1.65, 3.15)	0.941
rMBF	0.80(0.74, 0.84)	0.80(0.77, 0.84)	0.532
sMBF	1.88(1.32, 2.49)	1.95(1.32, 2.56)	0.794
SSS	3.00(1.00, 4.00)	3.00(2.00, 4.00)	0.887
SRS	0.00(0.00, 1.00)	0.00(0.00, 0.84)	0.184
SDS	2.00(1.00, 3.00)	2.00(1.00, 3.00)	0.602
CACS (AU)	571.98(337.93, 847.10)	555.62(372.39, 747.79)	0.858
FAI (HU)	-79.60 ± 6.81	-78.42 ± 6.69	0.300
Total Plaque Burden (%)	22.19(7.49, 53.10)	29.21(17.34, 62.02)	0.164
Calcified Plaque Burden (%)	3.24(0.45, 12.59)	3.79(0.80, 20.29)	0.406
Non-Calcified Burden (%)	18.37(6.54, 40.05)	27.43(10.38, 44.36)	0.156
Lipid Plaque Burden (%)	0.96(0.17, 2.75)	1.36(0.48, 3.87)	0.126

Fibrous Fatty Burden (%)	5.98(1.89,14.25)	9.56(2.50, 17.38)	0.159
Fibrotic Plaque Burden (%)	10.04(3.62, 22.29)	13.81(6.14, 25.52)	0.238
Positive Remodeling	136(85.0%)	42(93.3%)	0.144
Low Attenuation Plaque	137(85.6%)	43(95.6%)	0.072
Napkin-ring Sign	76(47.5%)	27(60.0%)	0.138
Spotty Calcification	106(66.3%)	34(75.6%)	0.236
MACEs	14(8.8%)	5(11.1%)	0.629
